# 基于低共熔溶剂和改进针式滤头的液膜微萃取结合高效液相色谱法测定牛奶中磺胺与氟喹诺酮残留

**DOI:** 10.3724/SP.J.1123.2025.03012

**Published:** 2026-04-08

**Authors:** Ruiming XU, Youyi WU, Zhihao GU, Wei WEI, Xingyu LU, Zihan XIA

**Affiliations:** 1.苏州科技大学环境科学与工程学院，江苏 苏州 215009; 1. School of Environmental Science and Engineering，Suzhou University of Science and Technology，Suzhou 215009，China; 2.江苏省环境科学与工程重点实验室，江苏 苏州 215009; 2. Jiangsu Key Laboratory of Environmental Science and Engineering，Suzhou 215009，China

**Keywords:** 低共熔溶剂, 针式滤头, 液膜微萃取, 抗生素残留, 牛奶样品, deep eutectic solvent （DES）, improved needle filter （INF）, liquid-membrane microextraction （LMM）, antibiotic residues, milk samples

## Abstract

乳制品中抗生素残留威胁食品安全，影响人体健康。为准确测定牛奶中磺胺（SAs）和氟喹诺酮（FQs）抗生素残留水平，本研究建立了基于低共熔溶剂（DES）和改进针式滤头-液膜微萃取（DES&INF-LMME）结合高效液相色谱（HPLC）检测牛奶样品中这两类抗生素残留的分析方法。筛选出辛酸为氢键供体、百里酚为氢键受体，合成疏水性DES作为膜萃取的萃取剂和支撑液膜（SLM）。进而考察了DES种类及用量、百里酚和辛酸的物质的量之比、萃取膜种类、样品体积、盐种类及添加量、pH值、转速和萃取时间等因素对萃取效率的影响，得到最佳萃取条件：DES（百里酚-辛酸（2∶1，*n/n*））用量为100 μL，采用聚四氟乙烯（PTFE）膜滤头，样品溶液体积为30 mL（添加3 g硫酸铵，调节溶液pH为7），萃取转速400 r/min，萃取时间35 min。在优化条件下，所开发DES&INF-LMME-HPLC方法的线性范围为2.86~1 000 μg/L（*r* ^2^>0.997 2），检出限为0.86~10.0 μg/L（*S/N*=3），定量限为2.86~33.3 μg/L（*S/N*=10）。富集倍数（EF）为32~84，回收率为99.4%~109.2%。在高、中、低加标水平下，日内、日间精密度分别≤4.9%和≤5.3%。该方法已成功应用于市售牛奶产品中4种抗生素的测定，在高、中、低3水平下4种抗生素的加标回收率为88.9%~113.4%。本方法准确、简便、灵敏，环境友好，可为乳制品中抗生素残留检测的新方法开发提供参考。

## Abstract (en)

Antibiotic residues in dairy products pose serious threats to food safety and affect human health. To accurately determine the level of sulfonamide and fluoroquinolone residues in milk， a deep eutectic solvent and improved needle filter-based liquid membrane microextraction （DES&INF-LMME） method combined with high performance liquid chromatography （HPLC） was developed for their determination in this study. Octanoic acid （hydrogen bond donor） and thymol （hydrogen bond acceptor） were selected to synthesize hydrophobic deep eutectic solvent， which acted as supporting liquid membrane （SLM） and extractant in the LMME process. Further investigations were conducted to examine the effects of various factors on extraction efficiency， including type and volume of DES， molar ratio of thymol to octanoic acid， type of extraction membrane， sample volume， salt type and addition amount， pH value， stirring speed and extraction time. The optimal extraction conditions were determined as follows： DES∶thymol-octanoic acid=2∶1 （*n/n*）， 100 μL； filter membrane， polytetrafluoroethylene （PTFE）； sample volume and pH， 30 mL （addition of 3 g ammonium sulfate） and pH=7； stirring speed， 400 r/min； extraction time， 35 min. Under the optimized conditions， the proposed DES&INF-LMME-HPLC exhibited good linearity in the range of 2.86-1 000 μg/L （*r* ^2^>0.997 2）， with limits of detection （LODs） （*S/N*=3） and limits of quantification （LOQs） （*S/N*=10） ranging from 0.86-10.0 μg/L and 2.86-33.3 μg/L， respectively. The obtained enrichment factors （EFs） ranged from 32 to 84 with recoveries of 99.4%-109.2%. The intra-day and inter-day precisions at three spiked levels （low： 30 μg/L； medium： 50 μg/L； high： 100 μg/L）， expressed as relative standard deviations （RSDs）， were no more than 4.9% and 5.3%， respectively （*n*=6）. This method has been successfully applied to the determination of four antibiotics in commercially available milk products. The spiked recoveries for the four antibiotics at high， medium and low levels ranged from 88.9% to 113.4%. The proposed method is accurate， simple， sensitive and eco-friendly， and could be a good reference to the development of new analytical method for antibiotic residues in dairy products.

牛奶是使用最广泛的日常食品之一^［1］^。为了确保包括牛奶在内的牛乳制品的产量，人们常使用抗生素类兽药治疗乳畜中高发的乳腺炎等细菌感染性疾病^［2，3］^，磺胺（SAs）和氟喹诺酮（FQs）类抗生素因其能有效对抗革兰氏阳性和革兰氏阴性细菌，也被广泛应用于乳畜产品中^［4］^。但随着这些抗生素的大量使用甚至滥用，会出现细菌耐药性问题，使得抗生素效果降低甚至无效^［5］^。此外，乳制品中残留的抗生素还会威胁食品安全，诱发人体产生耐药性^［6］^，造成机体免疫力下降，引起人体过敏性休克，甚至产生“三致”病变等健康问题^［7］^。为此，已有不少国家/组织对牛奶中兽药残留制定了严格的标准，我国规定牛奶中SAs及FQs类抗生素的最大残留值（MRL）为100 μg/kg。因此，亟需开发准确、灵敏、简便、绿色的牛奶制品中抗生素残留检测方法。

目前，食品中SAs和FQs抗生素的检测方法有高效液相色谱法（HPLC）、液相色谱-串联质谱法^［8］^、电化学传感法^［9］^、荧光光谱法^［10］^、毛细管电泳法^［11］^等，其中高效液相色谱法因其灵敏度高、可靠性好、应用广泛而成为测定食品中抗生素的首选方法^［12］^。但由于食品基质复杂、抗生素残留浓度低，需要进行样品前处理以浓缩所需的分析物，同时消除基质成分干扰^［13］^。常见的食品中抗生素的样品前处理技术有固相萃取^［14］^、分散液液微萃取^［15］^、三相混合萃取^［16］^及膜萃取（中空纤维膜液相微萃取（HF-LPME）^［17］^、平行人工膜萃取（PALME）^［18］^）等，但仍存在使用的有机溶剂部分有毒、萃取剂种类少的问题，因此需要开发新型绿色溶剂以提高萃取效率并减轻其环境影响。

低共熔溶剂（DES）因具有易于制备、熔点低、蒸汽压低、绿色环保、热稳定性好、生物相容性好、可再生等诸多符合绿色化学准则的优点^［19，20］^，有望成为传统有毒有机溶剂很好的替代品。DES由氢键受体（HBA）和氢键供体（HBD）通过分子间氢键作用形成，它也可与目标化合物形成氢键，从而提高萃取的效率和稳定性^［21，22］^。为此，许多学者将DES与膜萃取技术相结合以期开发出更高效、简洁的萃取方法^［23，24］^。Shishov等^［24］^在平板膜上原位生成DES的支撑液膜（SLM），实现了对奶粉中双酚的经济、高效萃取，检出限（LOD）为0.3~0.5 μg/L。Dowlatshah等^［25］^将合成琼脂糖膜作为SLM载体，结合DES和自制微流控装置，成功开发了人尿中高效萃取磺胺类药物的方法，LOD为50~77 μg/L。针式滤头因内置过滤膜且成本低廉，具有作为液膜微萃取载体的可能，但到目前为止，尚未发现DES结合针式滤头液膜微萃取牛奶中抗生素药物的报道。

本研究采用自行加工的针式滤头作为液膜微萃取装置，结合制备的疏水性DES作为SLM（萃取剂），在优化各前处理条件的基础上，建立了基于低共熔溶剂和改进针式滤头液膜微萃取（DES&INF-LMME）结合HPLC检测4种抗生素残留的方法，并成功应用于市售牛奶样品的检测。

## 1 实验部分

### 1.1 仪器、试剂与材料

Waters ARC HPLC带PDA检测器（美国Waters）；Nicolet iS5傅里叶变换红外光谱仪（美国赛默飞世尔科技公司）；TGL-15 B高速离心机（上海安亭科技仪器厂）；HJ-4A多头磁力搅拌器（江苏科析仪器有限公司）；STE 101打磨机（Mobile Drill）；KQ-500E超声波清洗器（昆山市超声仪器有限公司）。

磺胺甲基嘧啶（SM1，99%）、磺胺甲恶唑（SMX，99%）、加替沙星（GAT，99%）、氟罗沙星（FLE，99%）购自上海阿拉丁试剂有限公司；己酸（99%）、辛酸（99%）、壬酸（99%）、己醇（99%）、辛醇（99%）、癸醇（99%）、香豆素（99%）、丙酮、三氯乙酸、薄荷醇（99%）、橙花醇（98%）、丙二醇（99%）、硫酸铵（>99%）、氯化钠（>99.5%）、硫酸钠（>99%）、磷酸氢二钾（AR）购自泰坦科技股份有限公司；百里酚（99%）购自麦克林化学试剂有限公司。聚丙烯（PP）滤头购自上海钦森实验仪器有限公司；尼龙（NY）、聚四氟乙烯（PTFE）、聚偏氟乙烯（PVDF）滤头购自天津津腾实验设备有限公司。

分别称取适量上述抗生素标品于容量瓶中，用甲醇定容配制成400 mg/L的单标储备液，并于4 ℃下冷藏备用。标准工作液由甲醇稀释而成。

### 1.2 DES的制备

分别准确称量物质的量之比为1∶2的百里酚与辛酸于50 mL圆底烧瓶中，70 ℃下加热搅拌直至获得澄清透明液体，将所得液体（即DES）转移至10 mL离心管中，在干燥封闭条件下储存备用。

### 1.3 牛奶样品制备

将适量体积牛奶移入离心管中，之后再加入与牛奶体积比为1∶10的15%三氯乙酸，手动振荡离心管数次后于8 000 r/min离心5 min，取上清液经0.22 μm滤膜过滤备用。

### 1.4 改进萃取滤头的制作

用打磨机小心打磨PTFE萃取滤头下端（保留中间支撑轴，对两边进行打磨），直至完全露出膜体。用适量铁丝缠绕制作后续萃取所需磁感铁丝备用。所得萃取滤头如图1所示。

**图1:**
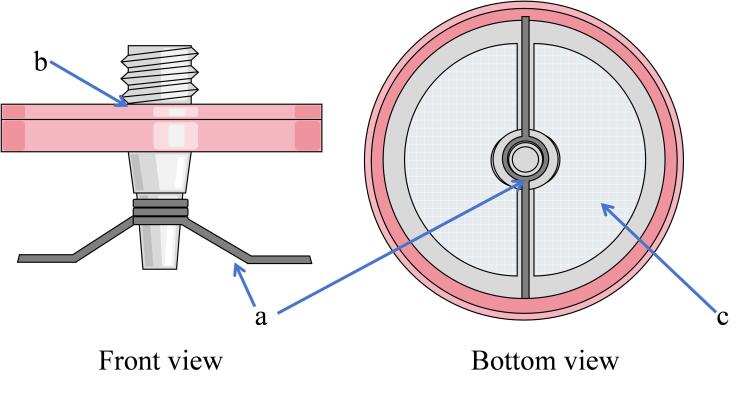
改进针式滤头示意图

### 1.5 样品萃取

萃取步骤如图2所示。将打磨好的PTFE萃取滤头放入丙酮中超声5 min清洁活化滤膜表面，在滤头底部固定磁感铁丝（图1）。在150 mL烧杯中移入处理过的30 mL牛奶样品，加入3 g 10%硫酸铵，完全溶解后调节溶液pH至7。准确移取100 μL DES，由滤头上端进口注入滤头当中，将带有DES的滤头露膜面浸入样品液中（滤头上端露出液面，图2），并于400 r/min磁力搅拌下萃取35 min，之后用微量进样针小心抽出DES，将100 μL甲醇注入改进滤头中，抽吸3次后与所得DES合并混合，待HPLC检测。

**图2:**
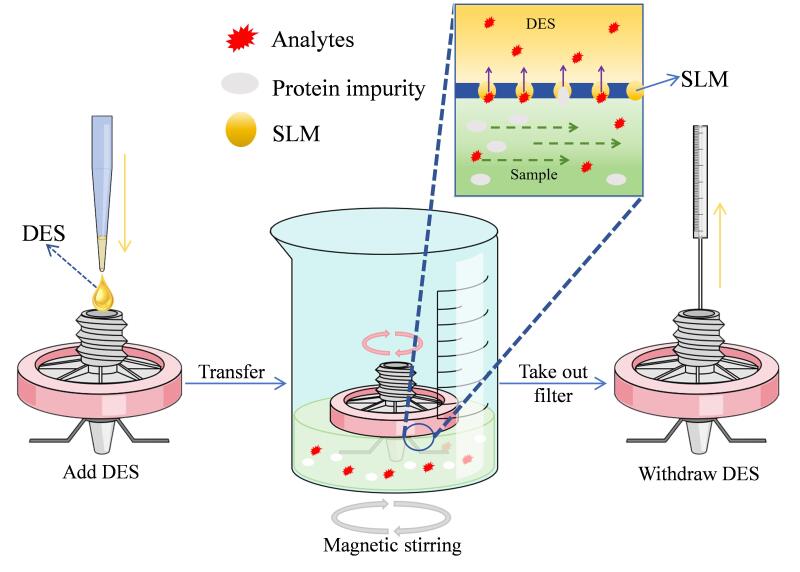
DES&INF-LMME方法示意图 SLM： support liquid film.

### 1.6 色谱条件

色谱柱：Agilent Eclipse XDB-C18色谱柱（150 mm×4.6 mm，3.5 μm）。流动相：0.1%甲酸水溶液（A）和甲醇（B）；检测波长：285 nm；柱温：30 ℃；进样量：5 μL；流速：0.8 mL/min；梯度洗脱程序：0~8 min，8%B~18%B；8~9 min，18%B~40%B；9~11.5 min，40%B~30%B；11.5~17.5 min，30%B；17.5~25 min， 30%B~8%B； 25~28 min，8%B。

## 2 结果与讨论

利用单因子优化法，分别考察了DES种类及用量、膜种类、样品体积及pH、盐种类及添加量、萃取时间和转速对牛奶样品中SAs及FQs萃取效率的影响。

### 2.1 DES的优化

作为SLM，DES对液膜微萃取效果至关重要。为此，本研究采用百里酚、有机酸、醇类等制备了14种疏水性DES（见表1）并考察其对牛奶样品中4种抗生素（SM1、FLE、SMX、GAT）萃取效果的影响，结果见图3a。可见，对于具有相同HBA、不同HBD的DES，脂肪酸比相同碳链长度的醇作为HBD萃取效率好。这可能是由于4种目标物都具有较强极性（辛醇-水分配系数log *P*为0.35~1.72），而脂肪酸比同链长的醇极性大，根据“极性相似相溶”原理，这更有利于提取极性强的目标物^［26］^。而保持脂肪酸不变时，百里酚作为HBA的DES萃取效果远优于薄荷醇，这是因为除了氢键作用外，百里酚的苯环与目标物苯环间还存在*π-π*相互作用。在百里酚和脂肪酸组成的DES中，DES-7的萃取效果最佳，因此在后续实验中选择百里酚和辛酸进行进一步优化。

**表1:** 14种DES的组成

DES	HBA	HBD1	HBD2	Molar ratio	Density/（g/mL）	Melting point/℃
DES-1	menthol	hexanoic acid	-	1∶2	0.9197	<-20
DES-2		heptanoic acid	-	1∶2	0.9308	<-20
DES-3		octanoic acid	-	1∶2	0.9326	4
DES-4		nerol	formic acid	1∶1∶2	0.9583	<-20
DES-5	thymol	hexanoic acid	-	1∶1	0.9674	<-20
DES-6		heptanoic acid	-	1∶1	0.9513	<-20
DES-7		octanoic acid	-	1∶1	0.9665	4
DES-8		decanoic acid	-	1∶1	0.9656	<-20
DES-9		hexanol	-	1∶2	0.8969	<-20
DES-10		octanol	-	1∶2	0.8961	<-20
DES-11		decyl alcohol	-	1∶2	0.8752	0
DES-12		propylene glycol	-	2∶1	0.9964	<-20
DES-13		nerol	-	1∶1	0.9541	<-20
DES-14		coumarin	-	2∶1	0.9811	<-20

HBA： hydrogen bond acceptor； HBD： hydrogen bond donor.

**图3:**
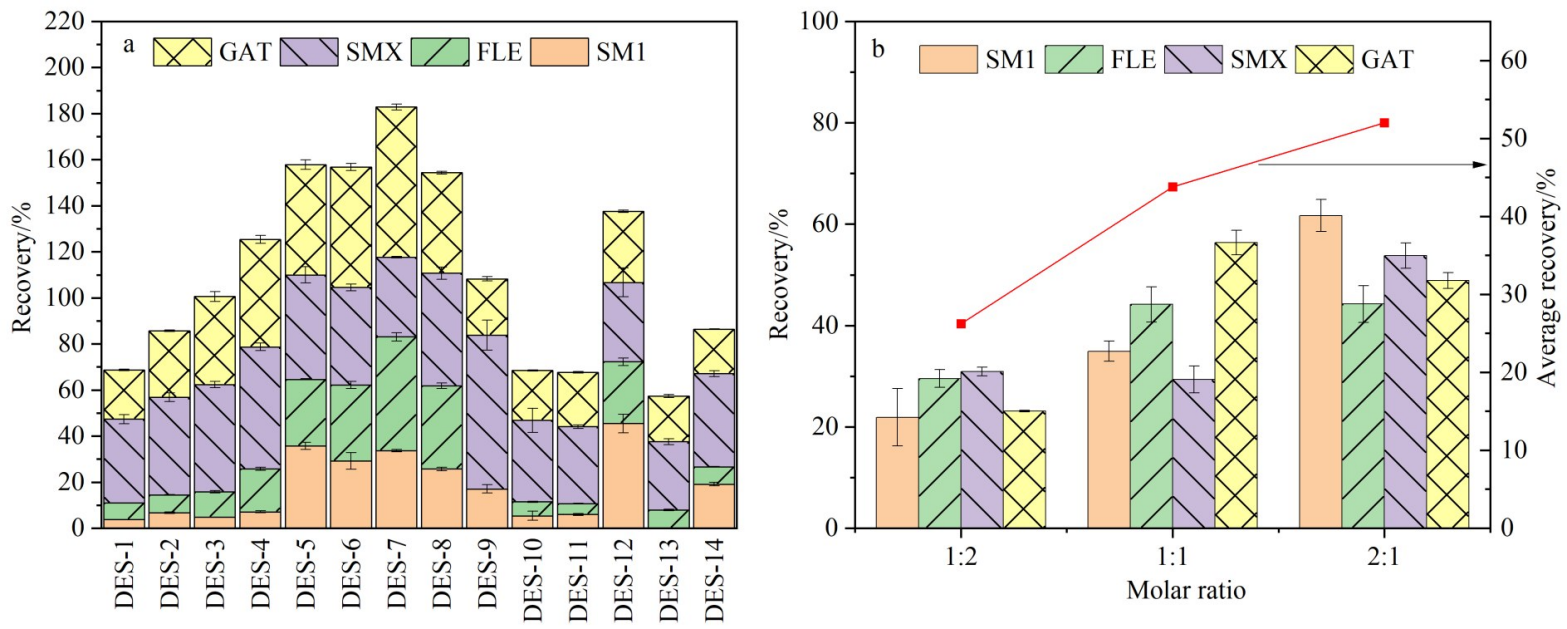
（a）DES种类、（b）百里酚与辛酸物质的量之比对萃取效率的影响（*n*=3）

考虑到HBA和HBD物质的量之比会对DES的理化性质产生影响，实验考察了不同百里酚与辛酸物质的量之比（1∶1、1∶2、2∶1）对目标物萃取效果的影响。由图3b可知，随着DES中百里酚占比的增加，萃取效率逐渐上升。当百里酚与辛酸的物质的量之比为2∶1时，目标物的萃取效率最大，因此后续实验采用百里酚-辛酸（2∶1，*n/n*）作为DES。

### 2.2 DES的表征

通过FT-IR表征了上述实验筛选出的DES。如图4所示，1 706、2 930 cm^-1^处的吸收峰分别为辛酸的-COOH上的羰基振动峰和饱和C-H振动峰；百里酚的C-H振动峰和O-H振动峰分别位于2 961 cm^-1^和3 440 cm^-1^；相对于辛酸、百里酚的红外谱图，DES在1 706 cm^-1^处的羰基振动峰变小了，且DES中O-H振动峰由3 440 cm^-1^（百里酚）移至3 400 cm^-1^处，表明辛酸与百里酚之间产生了氢键作用。此外，通过对比DES对目标物萃取前后红外光谱图，发现-COOH中羰基吸收峰峰强变弱，这可能是因为DES与目标物间产生了相互作用。

**图4:**
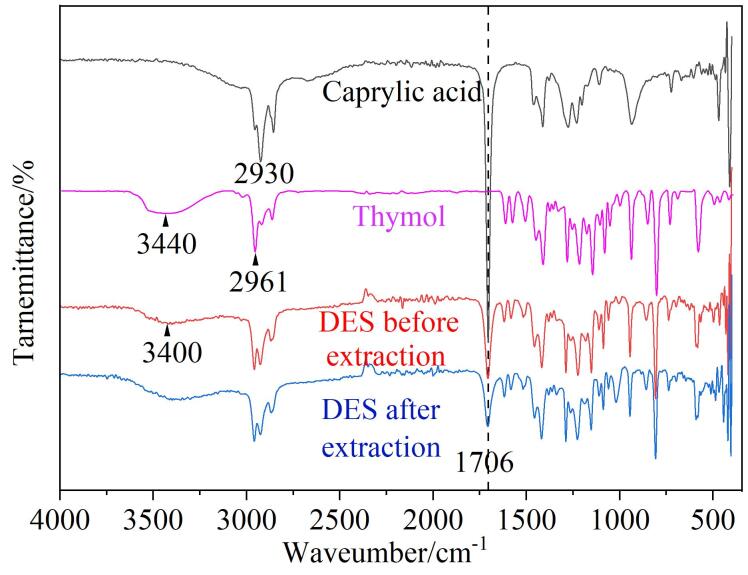
百里酚、辛酸和萃取前后DES的红外光谱

### 2.3 萃取条件的优化

#### 2.3.1 膜种类优化

膜种类是膜萃取方法的关键因素之一，不同种类的膜成分、机械强度、疏水性等不同，对目标物萃取效率也有差异^［18，27］^。基于此，本研究考察了NY、PP、PTFE、PVDF 4种膜材料对牛奶样品中目标物萃取效率的影响，结果如图5所示。可知，PP和PTFE膜对4种目标物萃取效果较好，PVDF膜次之。可能是因为PP和PTFE接触角大于90°，具有比PVDF和NY更强的疏水性，使得它们和疏水DES之间具有更高的亲和力，从而获得了更高的萃取效率。考虑到PP和PTFE膜的萃取效果差异小（平均回收率差<1%），但是PTFE膜相较于PP膜更加耐磨且化学惰性更好，最终选择PTFE作为滤头膜材料。

**图5:**
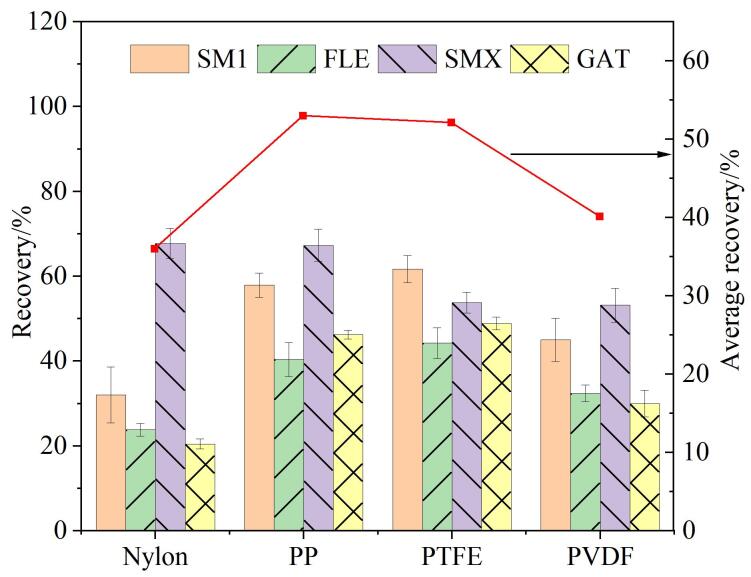
膜种类对萃取效率的影响（*n*=3）

#### 2.3.2 DES用量和样品体积优化

本实验探讨了不同DES体积（75、100、125、150、175 μL）对目标物萃取效果的影响，结果如图6a所示。当DES用量由75 μL增加到100 μL时，各目标物的富集倍数（EF）分别提高到原来的1.01~1.16倍；而DES用量超过100 μL时，目标物的富集倍数均有所下降。这是由于萃取接近平衡时，分析物的萃取量增加较小或不再增加，萃取剂的增加产生稀释效应^［28］^，造成萃取剂中目标物的浓度相对降低，因而EF降低。因此选择100 μL作为后续实验萃取剂的用量。

**图6:**
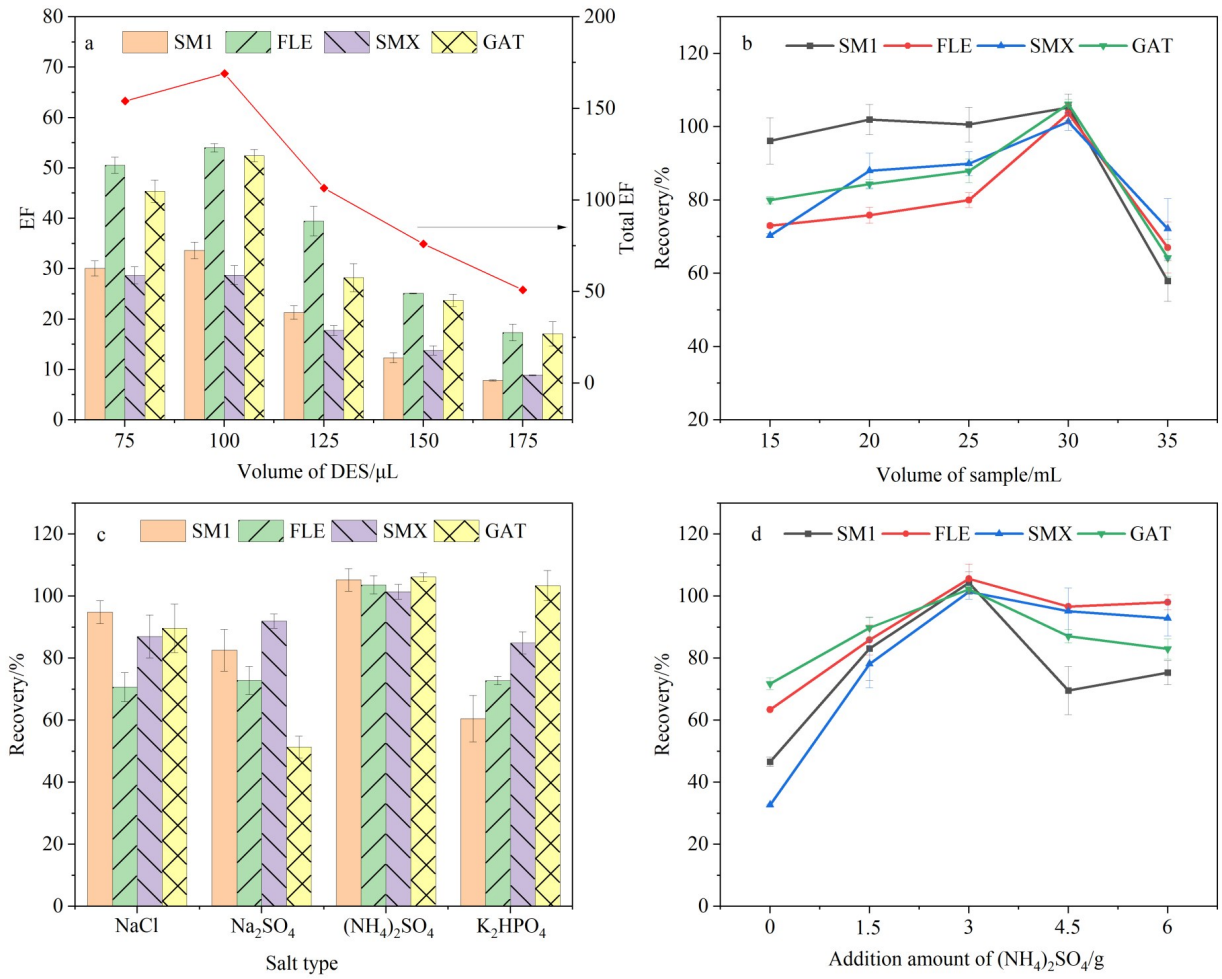
（a）DES用量、（b）样品体积、（c）盐种类和（d）盐添加量对萃取效率的影响（*n*=3）

样品体积也是影响萃取效果的重要因素之一，体积过小会使得萃取装置不易在液相中保持平衡，甚至因液面低于滤头膜面造成无法萃取，而体积过大也可能不利于萃取。因此，本文考察了样品体积（15、20、25、30、35 mL）对萃取效率的影响。如图6b所示，当样品体积由15 mL上升到30 mL时，4种目标物的回收率均有不同程度的提升，并于30 mL时达到最大值；样品体积继续增加时，除SMX峰面积缓慢降低外，其余目标物萃取效果均明显下降。此时，由于样品对滤头的阻力过大，改进的滤头萃取的分析物通量变小，造成萃取效率降低。因此，后续实验采用样品体积为30 mL。

#### 2.3.3 盐种类和添加量对萃取效率的影响

盐析效应可以降低分析物在给出相（样品）的溶解度，提高目标物在接受相的分配，达到提高萃取效率的目的^［29］^。本实验考察了4种（氯化钠、硫酸钠、硫酸铵和磷酸氢二钾）不同的盐对目标物萃取效果的影响。结果表明：使用硫酸铵时，4种目标物均取得了最佳萃取效果，而使用硫酸钠的效果最差（图6c）。同时，盐添加量也会引起样品溶液离子强度、黏度等发生变化，进而影响盐析效应。因此，本研究进一步考察了不同添加量的硫酸铵（0、1.5、3、4.5、6 g）对目标物萃取效果的影响。结果表明：4个目标物的回收率随着硫酸铵添加量的增加而增加，在3 g时达到最大，随后，目标物回收率均有所下降（图6d）。这可能是因为盐浓度过高，样品溶液黏度增大，降低了目标物向萃取剂的传质效率^［30］^。

#### 2.3.4 样品pH值对萃取效率的影响

SAs和FQs都是可电离两性化合物，pH值的改变能够影响其在样品溶液中的存在形式，进而影响其萃取富集^［6，31］^。为此，本工作考察了不同pH值（3~9）的样品溶液对目标物富集倍数的影响。如图7a所示，当pH=4时，SAs目标物达到最大EF，而FQs目标物在pH=7时达到最大，这与根据SAs和FQs的解离常数p*K*_a_估算的它们呈中性分子的pH值接近，而中性目标物分子更易于被萃取^［32］^。相比于pH=4，pH=7时4种目标物的平均EF增加了5.01。当pH值由7逐渐上升至9时，观察到萃取过程样品溶液中出现乳状悬浮物，4种目标物的EF均大幅下降。因而后续实验将样品溶液pH值调至7。

**图7:**
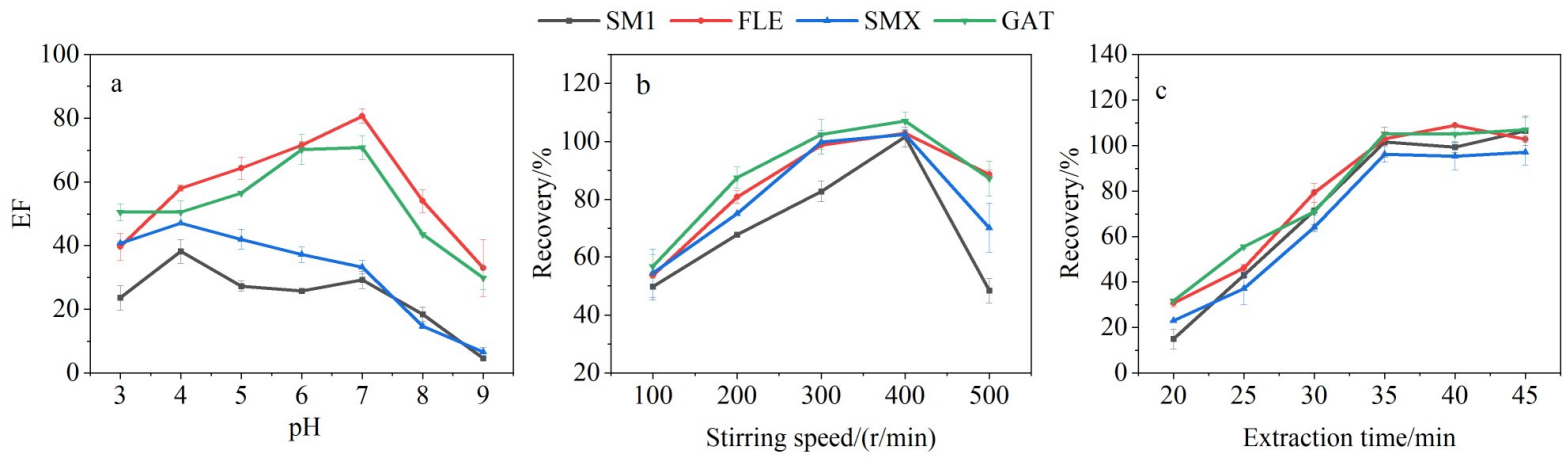
（a）样品溶液pH值、（b）搅拌速度及（c）萃取时间对萃取效率的影响（*n*=3）

#### 2.3.5 转速和萃取时间优化

在膜萃取过程中，转速是影响萃取效率的重要因素，适当的搅拌速度能够加速目标物质的扩散而缩短萃取动态平衡的时间^［33］^，但过快的速度可能会导致SLM脱落而造成萃取剂在样品溶液中逸散或引入气泡阻隔样品与SLM之间的传质。为此，本实验考察了不同转速（100~500 r/min）对目标物萃取效率的影响。如图7b所示，转速为400 r/min时，4种目标物回收率达到最大。

本研究进而考察了不同萃取时间（20~45 min）对目标物萃取的影响，如图7c所示。结果发现，35 min萃取效果最佳且延长时间无明显变化，表明此时目标物的萃取基本达到平衡。

### 2.4 基质效应

以不同品牌空白牛奶样品为基质，按1.5节方法进行前处理，用空白基质萃取液配制5个不同质量浓度的系列混合标准溶液（0.05、0.1、0.2、0.5、1 µg/mL），另用纯水配制相同质量浓度的溶剂系列混合标准溶液，以基质标准曲线和溶剂标准曲线的斜率比对基质效应（ME）进行评价。当ME<1时为基质抑制效应；当ME>1时为基质增强效应；当0.8≤ME≤1.2时为弱基质效应^［34］^。结果表明，所得4种目标物的ME为0.86~1.07（表2），说明经前处理后的牛奶基质对所建方法的测定结果影响较小，证明了本方法在测定真实牛奶样品中SAs和FQs残留的可行性。

**表2:** 4种目标分析物的基质效应

Analyte	MEs			
	Sample 1	Sample 2	Sample 3	Sample 4
SM1	0.86	0.89	0.94	0.96
FLE	0.90	0.94	0.88	0.98
SMX	0.96	0.92	1.02	1.02
GAT	0.99	1.07	1.05	1.04

### 2.5 方法学验证

#### 2.5.1 线性范围、检出限、定量限和富集倍数

配制系列混合标准工作溶液，在最佳优化条件下萃取目标物后进行HPLC分析，以目标物质量浓度为横坐标，对应峰面积为纵坐标，绘制工作曲线。如表3所示，方法的线性范围为2.86~1 000 μg/L（相关系数*r* ^2^>0.997 2）。根据仪器软件自带信噪比（*S/N*）计算功能，在3倍和10倍*S/N*下，LOD和定量限（LOQ）分别为0.86~10 μg/L和2.86~33 μg/L。对空白加标牛奶样品（100 μg/L）进行分析，计算各分析物富集后的含量，并与加标水平进行比较，计算出各分析物的EF为32~84。

**表3:** DES&INF-LMME方法的分析性能

Analyte	Linear range/（μg/L）	*r* ^2^	LOD/（μg/L）	LOQ/（μg/L）	Recovery/%	EF	Intra-RSDs/% （*n*=6）			Inter-RSDs/% （*n*=6）		
							30 μg/L	50 μg/L	100 μg/L	30 μg/L	50 μg/L	100 μg/L
SM1	13.8‒1000	0.9972	4.13	13.8	109.2	36	2.6	4.9	1.4	4.1	5.2	5.0
SMX	33.3‒1000	0.9981	10.0	33.3	99.4	32	3.4	2.6	4.3	4.0	4.6	4.2
FLE	2.86‒1000	0.9990	0.86	2.86	102.6	84	4.2	1.6	3.1	3.1	4.2	1.3
GAT	15.4‒1000	0.9973	4.62	15.4	103.3	71	0.9	3.6	3.6	3.2	4.8	5.3

*r* ^2^： coefficient of determination.

#### 2.5.2 回收率与精密度

在空白牛奶样品中分别进行三水平加标（30、50和100 μg/L）试验，并在最佳优化条件下进行加标回收试验。每个加标水平在1 d内平行测定6次以计算日内精密度；每个加标水平连续测定6 d以计算日间精密度。结果显示，所得日内、日间RSD均≤5.3%，表明该方法具有良好的精密度。加标30 μg/L时，牛奶样品的回收率为99.4%~109.2%（表3），表明所建立的方法准确度较高。

### 2.6 实际样品检测

将该方法应用于4个市售品牌牛奶样品中4种SAs及FQs的检测（表4），样品中均未检出上述抗生素残留。进一步的加标实验表明：在3个加标水平（30、50和100 μg/L）下，4种抗生素药物的回收率为88.9%~113.4%，RSD≤6.5%。图8是采用本方法检测的上述牛奶及其加标样品的色谱图。

**表4:** 真实的牛奶样品中4种目标分析物的加标回收率（*n*=3）

Analyte	Spiked/（μg/L）	Sample 1			Sample 2			Sample 3			Sample 4		
		Found/（μg/L）	RE/%	RSD/%	Found/（μg/L）	RE/%	RSD/%	Found/（μg/L）	RE/%	RSD/%	Found/（μg/L）	RE/%	RSD/%
SM1	0	-	ND	-	-	ND	-	-	ND	-	-	ND	-
	30	32.8	109.2	0.9	31.5	105.1	6.5	34.0	113.4	5.4	28.8	95.9	4.4
	50	53.1	106.3	6.1	44.5	88.9	0.6	51.1	102.2	4.2	51.9	103.8	6.5
	100	91.8	91.8	3.6	95.3	95.3	3.3	101.9	101.9	5.9	91.4	91.4	0.3
Fle	0	-	ND	-	-	ND	-	-	ND	-	-	ND	-
	30	30.8	102.6	2.0	31.1	103.8	3.0	32.4	107.9	5.9	26.7	88.9	2.4
	50	50.5	101.1	3.8	50.6	101.2	1.1	54.4	108.8	3.6	48.4	96.8	0.8
	100	104.1	104.1	3.5	107.3	107.3	4.3	111.3	111.3	2.8	91.2	91.2	1.7
SMX	0	-	ND	-	-	ND	-	-	ND	-	-	ND	-
	30	29.8	99.4	1.8	30.9	103.3	6.0	28.6	95.4	2.1	30.7	102.4	6.2
	50	52.3	104.7	4.1	49.6	99.3	1.7	46.8	93.6	2.9	54.9	109.8	2.8
	100	99.2	99.2	4.2	101.7	101.7	1.5	100.9	100.9	5.6	94.4	94.4	1.5
GAT	0	-	ND	-	-	ND	-	-	ND	-	-	ND	-
	30	31.0	103.3	2.6	30.9	103.1	0.5	30.9	103.2	4.6	30.9	102.9	0.7
	50	52.8	105.6	0.5	50.8	101.5	3.8	51.8	103.7	2.2	46.4	92.8	3.5
	100	103.7	103.7	5.5	104.3	104.3	2.7	107.3	4.5	96.5	96.5	96.5	4.5

RE： recovery. ND： not detected.

**图8:**
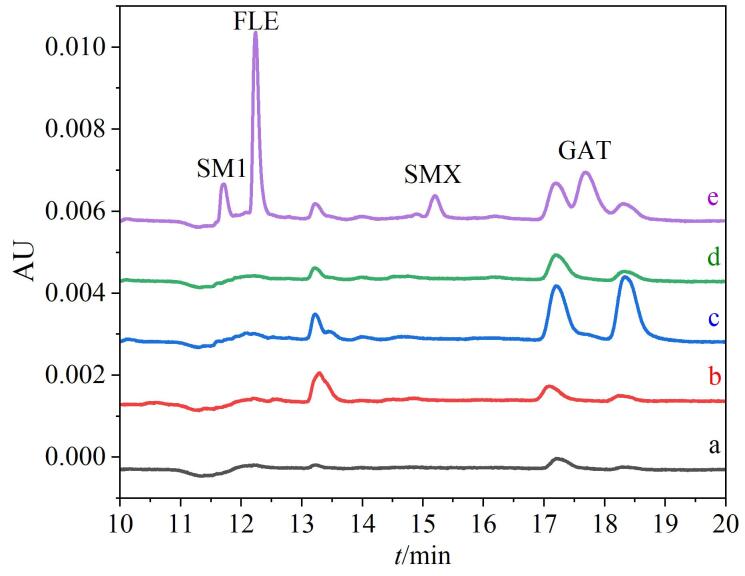
真实牛奶样品的色谱图

### 2.7 方法比较及绿色度、可行性评价

与其他方法比较（见表5），本方法所得LOD略高于液相色谱-串联质谱方法^［35，36］^，但比其他方法^［11，25，37，38］^更灵敏。本方法与其他方法的回收率相当。与DLLME方法^［11，37］^相比，本方法无需离心，前处理步骤更为简洁；并且，本方法改进滤头制作简单，仅需由滤头上端加入和抽出萃取相，操作难度小于中空纤维-液相微萃取等方法，且无需大型耗能萃取设备。相较于使用有机溶剂乙腈的方法^［35，36］^，本方法使用无毒的DES作为萃取剂（SLM），对环境更为友好。

**表5:** 本方法与其他方法的比较

Method	Sample	Analytes	Extraction solvent	Linear range	LOD	Recovery/%	Ref.
In-situ DES-LPME-HPLC-FLD	meat	FLE	DES （200 μL）	40-4000 μg/kg	15 μg/kg	98.0-108.0	［^38^］
VA-DLLME-DES-UPLC-MS	honey	FLE	DES （150 μL）	2-100 μg/L	3 μg/kg	84.9-100.1	［^37^］
QuEChERS-UHPLC-MS/MS	soil	FLE， SM1， SMX	acetonitrile （20 mL）	1-200 μg/L	2-5 μg/kg	56.6-120.2	［^35^］
SO-DLLME-DES-BE-MECC	milk	GAT	DES （200 μL）	30-4800 μg/L	10 μg/L	96.5-104.9	［^11^］
MMF-SPME-HPLC	milk	SMX， SM1	DES@MOF/MIP （desorption： 2 mL acetonitrile）	5-400 μg/L	1.4-2.1 μg/L	94.7-99.7	［^36^］
DES-AM-MLPME-HPLC	urine	SMX， SM1	DES as SLM （2 μL）	150-10000 μg/L	50-77 μg/L	88.0-96.0	［^25^］
DES&INF-LMME-HPLC	milk	SM1， SMX， FLE， GAT	DES （100 μL）	2.86-1000 μg/L	0.86-10 μg/L	88.9-113.4	this work

FLD： fluorometric detection； VA： vortex-assisted； SO： salting out； MECC： micellar electrokinetic capillary chromatography； MMF： multiple monolithic fiber； AM： agarose membranes； MOF/MIP： metal-organic framework /molecularly imprinted polymers.

为进一步评价方法的环境影响，本文采用Complex-GAPI和AGREE评价了本方法的绿色度，其中Complex-GAPI绿色评价程序是从样品采集与运输存储、样品的制备、溶剂及化合物的使用、仪器能耗、方法种类5个部分15个方面进行评估（图9a），除此之外，还增设了对DES合成过程的绿度评价；而AGREE程序则将样品前处理内容分成12小块（图9b）。分别用绿、黄、红3个颜色评价各小块因素对环境的影响程度，绿色代表环境影响小、黄色代表中等、红色代表环境影响大。如图9a所示，本方法14个方面（14/15）为绿色或黄色，表明方法较为绿色，DES合成程序评价栏还获得了0的评分（评分越低越绿色）。采用AGREE对本方法的绿度进行评价所得结果为0.63（图9b），再次证明所建立的方法较为绿色。此外，还使用BAGI星图对所建方法的实用性进行评估（象形图中深蓝色、蓝色、浅蓝色和白色分别对应高、中、低和不符合设定的标准，所得分数越高实用性越高）。结果显示，本方法所得BAGI分数为60（图9c），表明该方法具备一定的实用性和可推广价值。总之，该方法具有准确可靠、操作简单、节省试剂、绿色等优点。

**图9:**
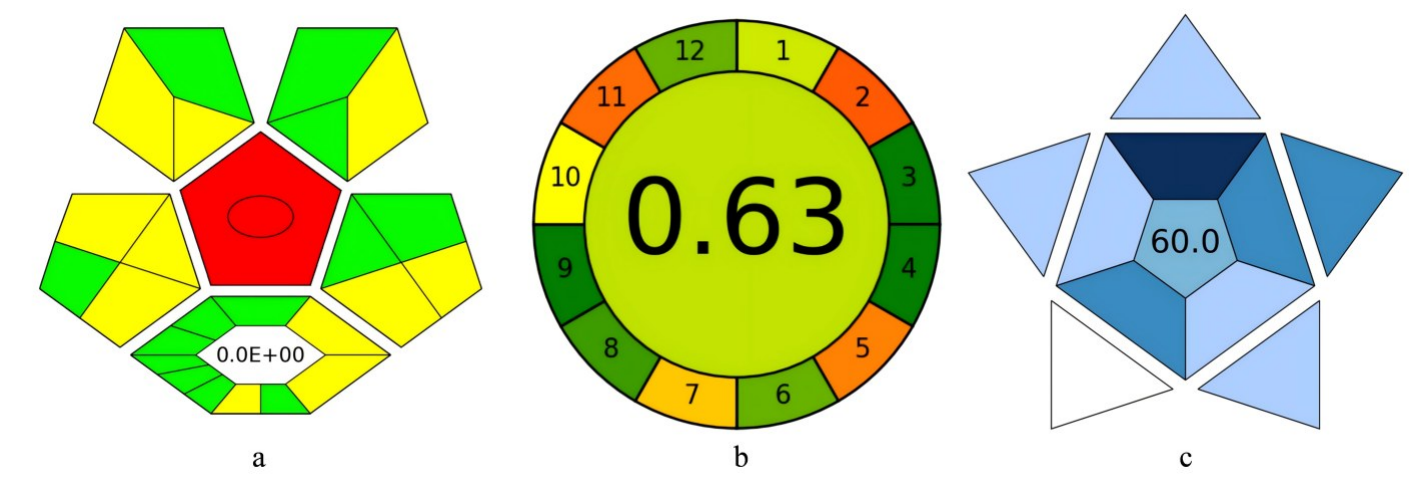
DES&NFH-LMME程序（a、b）绿色度及（c）实用性评价

## 3 结论

本文建立了基于DES和改进针式滤头的液膜微萃取结合HPLC测定牛奶中4种SAs和FQs抗生素残留的方法，并成功应用于实际样品的分析。本工作改进了针式滤头并将其作为液膜微萃取载体，选用绿色溶剂DES作为萃取剂和SLM，简化了萃取和操作程序（省去了传统DLLME法的离心和过滤两个步骤），并通过专业评价软件对本方法绿度和实用性进行了评价。本方法测定准确可靠，操作简单，绿色环保，未来可实现高通量萃取，可应用于乳制食品源中抗生素残留的检测，并可为抗生素残留检测新方法的开发提供参考。本方法开发装置自动化、小型化程度尚有提升空间，今后可进一步改进萃取滤头装置的制作，开发相应的高通量自动萃取装置。
